# Red Wolf (*Canis rufus*) Recovery: A Review with Suggestions for Future Research

**DOI:** 10.3390/ani3030722

**Published:** 2013-08-13

**Authors:** Joseph W. Hinton, Michael J. Chamberlain, David R. Rabon

**Affiliations:** 1 Warnell School of Forestry and Natural Resources, University of Georgia, Athens, GA 30602, USA; E-Mail: mchamberlain@warnell.uga.edu; 2 Red Wolf Recovery Program, United States Fish and Wildlife Service, P.O. Box 1969, Manteo, NC 27954, USA; E-Mail: david_rabon@fws.gov

**Keywords:** *Canis rufus*, *Canis latrans*, conservation, coyote, demographics, hybridization, inbreeding, red wolf

## Abstract

**Simple Summary:**

Once widespread in the Eastern United States, early 20th century predator-control programs reduced red wolves to a remnant population by the 1970s. The U.S. Fish and Wildlife Service, through the Red Wolf Recovery Program, restored red wolves to northeastern North Carolina in 1987. After 25 years of restoration efforts, issues of hybridization with coyotes, inbreeding, and human-caused mortality continue to hamper red wolf recovery. To understand how these issues influence recovery efforts, we examine the history of red wolf restoration and its challenges. We then formulate areas of research that are of direct relevance to the restoration of red wolves.

**Abstract:**

By the 1970s, government-supported eradication campaigns reduced red wolves to a remnant population of less than 100 individuals on the southern border of Texas and Louisiana. Restoration efforts in the region were deemed unpromising because of predator-control programs and hybridization with coyotes. The U.S. Fish and Wildlife Service (USFWS) removed the last remaining red wolves from the wild and placed them in a captive-breeding program. In 1980, the USFWS declared red wolves extinct in the wild. During 1987, the USFWS, through the Red Wolf Recovery Program, reintroduced red wolves into northeastern North Carolina. Although restoration efforts have established a population of approximately 70–80 red wolves in the wild, issues of hybridization with coyotes, inbreeding, and human-caused mortality continue to hamper red wolf recovery. We explore these three challenges and, within each challenge, we illustrate how research can be used to resolve problems associated with red wolf-coyote interactions, effects of inbreeding, and demographic responses to human-caused mortality. We hope this illustrates the utility of research to advance restoration of red wolves.

## 1. Introduction

**Figure 1:**
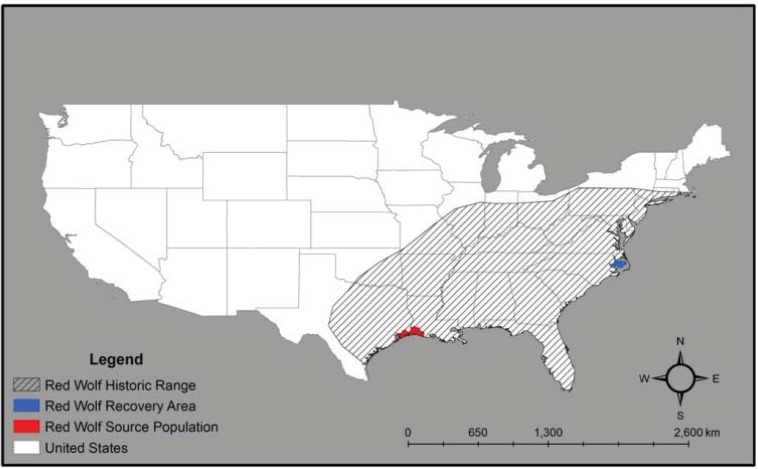
Historic and current range of red wolves (*Canis rufus*) in North America.

Perceived threats to human enterprise have historically motivated efforts to exterminate large carnivores such as wolves, bears, and lions. In particular, wolves have been extirpated from much of their historical ranges in North America by government-supported eradication campaigns protecting agricultural and livestock interests. However, changes in American societal beliefs have resulted in profound changes to how wolves are perceived. The passage of the Endangered Species Act of 1973 (ESA) paved the way for restoration of wolf populations that were severely reduced or extirpated during the 19th and early 20th centuries. When the ESA was legislated, gray wolves (*Canis lupus*) and red wolves (*Canis rufus*) existed as declining remnant populations in the contiguous United States. Although gray wolf populations in Alaska and Canada were stable and the species was not threatened with extinction, red wolves were afforded no refuge. Red wolves were likely the first New World wolf species to come in contact with Europeans and, consequently, the first to be persecuted. Prior to European colonization, red wolves were common in the Eastern United States and they inhabited an area from the Atlantic coast west to central Texas, with the Ohio River Valley, Northern Pennsylvania, and Southern New York being its northernmost range and their distribution extending south to the Gulf of Mexico (Figure 1) [1,2]. At the turn of the 20th century, red wolves were extirpated throughout most of their range and approximately 100 individuals occupied coastal habitats of Eastern Texas and Western Louisiana [3,4]. Declining because of aggressive predator-control programs and surrounded by an expanding coyote (*Canis latrans*) population, red wolves were incapable of maintaining self-sustaining populations. They began hybridizing with coyotes when they were unable to find conspecific mates and canid populations in the region gradually became genetically admixed [3,5,6]. This generated concerns that the last remaining red wolves would be genetically assimilated into the coyote genome through hybridization, so the Southeast Texas and Southwest Louisiana populations were targeted for restoration efforts [6].

After the passage of the ESA, the United States Fish & Wildlife Services (USFWS) established the Red Wolf Recovery Program (Recovery Program) with the task of locating and preserving populations of red wolves in southeast Texas and southwest Louisiana [7]. However, with rapidly declining red wolf populations and extensive hybridization, the USFWS decided to remove the last red wolves from the wild and place them in captivity. The Recovery Program’s objectives soon changed to capture as many red wolves as possible for propagation in captivity, and to re-establish red wolf populations within the species’ historic range in the near future [7]. To find pure red wolves for the captive-breeding program, the Recovery Program captured as many wild red wolf-like canids as possible in Southeast Texas and Southwest Louisiana. From 1973 through 1980, approximately 400 canids were captured and 43 met the morphological standards to be considered red wolves. Breeding experiments were then conducted with those 43 individuals and, eventually, 14 individuals met the criteria established to define the species. These individuals were used as the founders to begin the captive-breeding program [7]. The red wolf was declared extinct in the wild in 1980, becoming the first species to be purposely extirpated in the wild to save it from extinction. 

The captive-breeding program safeguarded the last remaining red wolves and served as the last repository of the red wolf genome. The primary objectives of the captive-breeding program were to certify the genetic purity of wild-caught red wolves, increase the number of red wolves in captivity, and maintain a captive red wolf population for re-establishment of the species in the wild [7]. Red wolves readily reproduced in captivity with the first captive-born litters produced during 1977. Early efforts in the captive-breeding program then focused on developing procedures and protocols to ship, handle, and breed red wolves within a network of zoo facilities [8]. To maintain integrity within the captive-breeding program, the USFWS developed a Species Survival Plan^®^ (SSP) that was accepted by the Association of Zoos & Aquariums (AZA) [7]. This ensured that the species would be preserved in captivity until a strategy was developed for reintroducing red wolves in the wild. To acclimate captive red wolves to wild conditions, the Recovery Program began conducting experimental releases of captive-born red wolves on island propagation sites such as Bulls Island of the Cape Romain National Wildlife Refuge in South Carolina. Bulls Island became one of three island propagation sites that allowed the Recovery Program to develop restoration techniques.

During 1984, Alligator River National Wildlife Refuge (ARNWR) was established on the Albemarle Peninsula of northeastern North Carolina (NENC) when the Prudential Insurance Company donated approximately 480 km² (48,000 ha) of land to the federal government [7]. This area was identified as the future reintroduction site for red wolves because the refuge contained suitable prey for red wolves, coyotes were absent on the landscape, no livestock were present, and the presence of humans was low. In 1987, the USFWS released eight captive-born red wolves (four male-female pairs) onto ARNWR to begin reintroduction efforts. Initially, mortality rates were high as captive-born wolves were hit by cars, drowned, succumbed to disease, or were attracted to townships [9]. As a result, early attempts to established red wolves on ARNWR were aggressive and resulted in the release of more than 60 red wolves from 1987 through 1994 [9]. Eventually, the NENC population transitioned from captive-born individuals to wild-born individuals and the release of captive-born adult wolves to augment the NENC population ceased. Currently, almost all red wolves in NENC are wild born. Periodically, island-born juveniles and captive-born pups fostered into wild litters are used to maintain genetic diversity and health of the wild population. By the mid-1990s, red wolves in the wild formed packs, maintained territories, and successfully bred, and the reintroduction marked the first successful reintroduction of a wolf species. It also marked the first successful attempt to reintroduce a large predator that was completely extirpated from the wild. 

The USFWS initiated a second reintroduction in the Great Smoky Mountains National Park (GSMNP) of the southern Appalachians [10]. During 1991, the initial stage of the GSMNP reintroduction was implemented to gather information on interactions of red wolves and coyotes, livestock, and humans [11]. Initial efforts appeared successful when a mated adult pair and two pups established a territory in Cades Cove of the GSMNP, so the USFWS proceeded with a full-scale reintroduction. However, most of the 37 red wolves released were unable to establish and maintain territories within the park boundaries and left for better habitat on surrounding lower-elevation agricultural land [12]. Additionally, red wolves that maintained territories on GSMNP had low pup survival as a result of Parvovirus, malnutrition, and parasites [12]. After repeated introduction attempts and low pup survival, it was determined that the red wolf population on GSMNP would have to be perpetually managed within the park and the GSMNP red wolf reintroduction was terminated in 1998. Red wolves that remained in the park were subsequently captured and relocated to ARNWR. 

**Figure 2:**
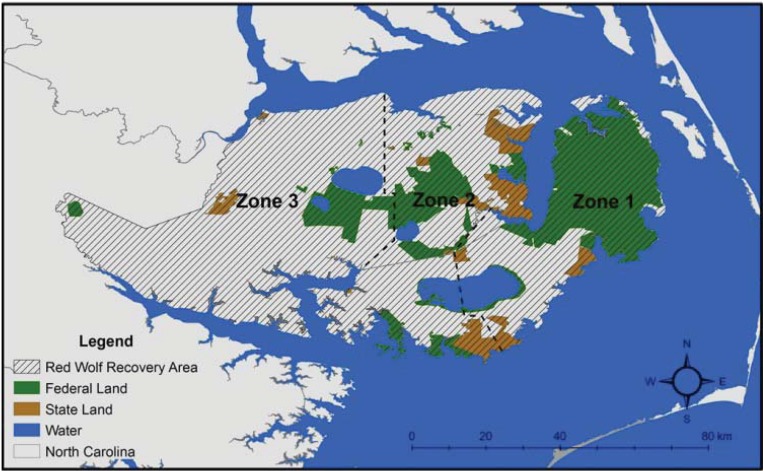
Management zone boundaries within the Red Wolf Recovery Area of northeastern North Carolina.

Although nearly 25 years have elapsed since red wolves were reintroduced into the wild, more than half of the red wolf population still exists in captivity. The captive-breeding program safeguards approximately 200 red wolves in more than 40 captive facilities around the United States while the reintroduced red wolf population has expanded throughout the Albemarle Peninsula to about 70–80 animals in approximately 15 packs [13]. Since 1987, the Recovery Area has expanded to accommodate the growing population from approximately 480 km² to approximately 6800 km² of federal, state, and private lands (Figure 2). Although red wolf restoration has experienced success in many ways, efforts to maintain the NENC population and to find future reintroduction sites continually face challenges. For instance, the red wolf species continues to be plagued by taxonomic controversy regarding its origin and arguments against the systematic validity of the red wolf have been used to oppose red wolf restoration [14,15]. Red wolves still remain a remnant population and experience a series of ecological threats such as hybridization with coyotes and inbreeding [16,17,18]. Without management of coyotes in the Recovery Area, it is likely that the red wolf population would be genetically assimilated into the eastern coyote population [16]. Additionally, the small number of red wolves makes the population in NENC susceptible to genetic drift and inbreeding depression [19]. To prevent inbreeding and maintain genetic diversity in the wild population, captive-born and island-born individuals are periodically released into the Recovery Area. Additionally, quixotic fervor within the hunting community to suppress predators continues to hamper red wolf population growth in NENC. Increased mortality by gunshot during the hunting season has reduced the number of red wolf packs, lowered red wolf survival, and has facilitated coyote expansion into the Recovery Area [18].

In the progress of overcoming these challenges to restoring red wolves to the wild, there is a need to consolidate knowledge and contemplate those experiences as recovery efforts move forward. Therefore, our objective is to provide a synopsis of the challenges to restoration of the red wolf and suggest future research needed to pursue full recovery of the species.

## 2. Red Wolf Taxonomy

Currently, scientists find themselves in a contentious debate regarding the taxonomy of New World wolves and its implications on the evolution, ecology, and conservation of *Canis* species in Eastern North America [20]. The origin of the red wolf is central to this debate [1,21,22,23]. Although scientific synthesis has led to new insights into the evolution and ecology of New World wolves, massive loss of historic and geographic genetic data and recent genetic introgression by coyotes continues to hinder consensus on red wolf origin [20]. Despite significant voids in data to adequately characterize the historic red wolf populations in the Southeast, limited and anecdotal data does exist to indicate the existence of a large canid in the Southeastern United States. 

The unique presence of a southeastern wolf was noted during the 18th century [24,25,26] and, by 1851, the red wolf was given a valid scientific name [27]. During the turn of the 20th century, several authors recognized structural differences between gray and red wolves and initiated revisions of the red wolf's taxonomic status [28,29,30]. Eventually, Goldman [31,32] described red wolves as distinct from gray wolves and coyotes based on cranial and dental characters and consigned all wolves of the Southeast to one species, *C. rufus*. By the 1960s, federal and state agencies generally assumed that viable populations of red wolves existed in the Southeast despite a great deal of confusion about the species status. McCarley’s [4] taxonomic study of red wolves concluded that red wolves had been replaced by coyotes and red wolf/coyote hybrids in most areas of Eastern Texas, Arkansas, Louisiana, and Oklahoma. His work indicated that a few red wolf populations still existed in parts of Louisiana. After examining a number of *Canis* specimens from the Southeast, Paradiso [33,34], and Pimlott and Joslin [35] confirmed McCarley’s findings and brought attention to what were believed to be the last surviving red wolf populations on the Gulf Coast in Southeast Texas and Southwest Louisiana [8].

Nowak [1,21] investigated the taxonomy of *Canis* species of Eastern North America using discriminant function analysis to evaluate the characteristics of modern and paleontological *Canis* skulls [1,21] and dentition [1]. In doing so, he was able to differentiate gray wolves, red wolves, coyotes, and domestic dogs (*Canis familiaris*) into separate groups and postulated that red wolves evolved from a transitional form (*i.e.*, *Canis mosbachensis*) between a wolf-like coyote ancestor and the gray wolf. Nowak [21] found no evidence that gray wolves existed in the Southeastern United States. Despite widespread occurrence of domestic dog in the Southeast, Nowak [21] found no evidence of introgression from domestic dogs into the red wolf and coyote populations. The earliest red wolf specimens showed no statistical overlap with gray wolves, coyotes, or domestic dogs and had similar multivariate distribution as the red wolf specimens from the Pleistocene era. Specimens collected before 1930 indicated hybridization between red wolves and coyotes was uncommon where their ranges approached. However, specimens from the 1930s until the 1950s indicated hybridization with coyotes was occurring over large areas of the red wolf's southern range where coyotes were replacing red wolves. Nowak [1,21] suggested that hybridization between red wolves and coyotes began at the turn of the 20th century when anthropogenic factors destroyed ecological and behavioral isolation. Despite coyote introgression into the red wolf genome during the 20th century, Nowak [1] reported that the morphology of modern red wolves is predominately like *C. rufus* that persisted in the Eastern United States 10,000 years ago.

Although it had been suggested that red wolves were the result of coyote and gray wolf hybridization [36], the hypothesis of a hybrid origin did not receive much attention until applied molecular techniques became the primary means of evaluating red wolf taxonomy. Analyzing mitochondrial DNA (mtDNA), Wayne and Jenks [22] evaluated the genetic integrity of red wolves in the captive-breeding program and reported no unique genetic markers in red wolves that were distinct from gray wolves and coyotes. Therefore, they concluded that the red wolf is a hybrid form derived from gray wolves and coyotes. Similar conclusions were reached by a series of genetic papers examining red wolf mtDNA and nuclear DNA (nDNA) that accepted the premise that red wolves originated from hybridization events occurring between 250 to 13,000 years ago [37,38,39,40]. However, these conclusions have been contested in morphological [1,2,41,42] and molecular [20,23,43,44,45,46,47,48] studies.

Examining the origin and taxonomy of wolves in eastern Canada, Wilson *et al.* [23,46] reported that captive red wolves and eastern wolves (*Canis lycaon*) have mtDNA control sequences more closely related with coyotes, while exhibiting unique haplotypes not found in gray wolves and coyotes. Although these mtDNA sequences don't occur in western coyotes, they cluster among western coyote populations and Wilson *et al.* [23] attributed this as evidence that red wolves, eastern wolves, and coyotes share a recent common ancestor in the New World independent of gray wolves. Other studies have supported these conclusions [20,45,49] and these results appear to reconcile early observations that red wolves and coyotes approached one another in morphology [21,31]. Although the results of these studies indicate that red wolves are not of hybrid origin, Wilson *et al.* [23] proposed that the red wolf and eastern wolf are genetically close enough to be considered a single species under *C. lycaon*. The disagreement among these genetic studies stems in part from differing assumptions about the nature of the coyote-like mtDNA found in eastern and red wolves. Those that support a hybrid origin interpret the coyote-like mtDNA as being from coyotes, whereas those that support the hypothesis that red wolves, eastern wolves, and coyotes share a common ancestry interpret the coyote-like mtDNA as being eastern wolf in origin and a result of incomplete lineage sorting.

Significant gaps in the historic and geographic genetic data and recent hybridization makes it difficult to sort out the evolutionary history of red wolves. As a result, the taxonomy of North American wolves is complex and not without debate. Prior to and during European colonization of the Southeast, there appears to have been a small wolf species present and its modern equivalent may be the red wolf. It is also possible that red wolves are morphologically and genetically similar to coyotes because they fall within the species limits of the coyote clade [20]. Although the door is open for future taxonomic revision, the hybrid origin of red wolves is difficult to reconcile because gray wolves have historically been absent from the Southeastern United States and, until the mid-20th century, coyotes were absent from the region for over 10,000 years [1]. Additionally, there is no evidence of ongoing hybridization between gray wolves and coyotes that are currently sympatric [48,50]. Modern hybridization among *Canis* species in the East makes it difficult to sort out the evolutionary history of red wolves. Recent and developing studies demonstrate that the taxonomy of red wolves is complex and morphological and molecular studies of fossilized wolves from the southeast are essential to settling the debate over red wolf origin. 

## 3. Ecological Challenges

### 3.1. Red Wolf and Coyote Hybridization

Red wolves and coyotes exist as a panmictic population in NENC and hybridization provides an exceptionally tough set of problems for red wolf recovery. Understanding how red wolves interact with coyotes is an important issue, which could dictate the success of the reintroduction project. During 1999, the USFWS re-evaluated the red wolf recovery effort by organizing a Population and Habitat Viability Assessment workshop (PHVA) [16]. Introgression of coyote genes into the red wolf population was considered the principal threat to recovery efforts when it was discovered that hybridization could render the wild red wolf population unrecognizable within several generations [16,17]. As a result, priorities were identified and the PHVA called for approaches that would prevent hybridization and promote the growth of a self-sustaining population of red wolves in NENC. An adaptive management plan [51] was designed during the PHVA with the intent to provide the Recovery Program flexibility to modify management schemes and scientific studies as conditions and threats to red wolf recovery change.

As history has proven, coyote populations are too resilient to state and federal eradication programs and clearing the Albemarle Peninsula of coyotes poses an overwhelming challenge. Two management techniques were developed during the PHVA to prevent hybridization. Coyotes and hybrids captured by USFWS personnel within the Recovery Area are reproductively sterilized (hereafter sterilized) and used as space holders until red wolves move in and occupy those areas. Coyotes and hybrids are taken to a local veterinary clinic in which females and males are sterilized by tubal ligation and vasectomy, respectively. This process keeps the hormonal system intact and avoids disrupting breeding and territorial behavior. Sterilized animals are fitted with mortality-sensitive radio-collars, released, and monitored for the duration of their life. This allows the Recovery Program to collect relevant information on coyote space use, habitat selection, and interaction with red wolves while suppressing coyote reproduction. In the event that a red wolf pairs with a sterilized coyote, the pair cannot produce hybrid litters. Additionally, sterilized coyotes that maintain territories keep those spaces occupied and prevent fertile coyotes from establishing breeding pairs on the landscape.

As recommended during the PHVA, the Recovery Area was divided into three management zones in which management efforts varied in intensity to minimize hybridization on the landscape (Figure 2). The ultimate management goal is to ensure that all *Canis* breeding pairs within the Recovery Area are red wolves. To implement this, Recovery Program biologists began eradicating coyotes and hybrids from Zone 1 while selectively using sterilized coyotes as space holders in Zone 2. When objectives in Zone 1 were completed, management efforts shifted west to Zone 2 in which sterilized space holders were removed to create space for red wolves. Once coyotes and hybrids were removed from Zone 1 and 2, management efforts would be undertaken in Zone 3. Implementing management goals in order of priority allowed the Recovery Program to minimize hybridization by monitoring red wolf and coyote packs throughout the Recovery Area and replacing coyotes and hybrids with red wolves when opportunities arose.

Prior to the PHVA, the Recovery Program assumed all canids captured within the Recovery Area were wolves unless animals were unusually small and coyote-like in appearance [17]. Once hybridization was considered the primary threat to recovery efforts, molecular techniques were developed to identify coyotes and hybrids and quantify introgression into the red wolf population. Using microsatellite markers from the 14 founding individuals and other captive red wolves to generate allele frequencies, a pedigree of the red wolf population was developed [52,53]. Animals are now blood sampled upon capture and identified as red wolves, coyotes, or hybrids using 17 microsatellite markers. As these methods were developed, a hybridization event that occurred during 1993 between a female red wolf and a male coyote was detected [18,53]. Individuals in the wild population considered red wolves were then correctly identified as 2nd and 3rd generation backcrosses from the male hybrid offspring of the 1993 hybridization event. When it was realized that removing all red wolves with introgression would essentially extirpate the wild red wolf population, the Recovery Program opted to allow wild reproduction among red wolves to slowly breed the coyote genetics out. To accelerate purging of coyote genetics, the Recovery Program selectively culled animals they thought were not red wolves. Over time, selective management of backcrosses and minimizing hybridization has been successful in limiting coyote introgression in the wild red wolf population to less than 5% in 2006 [53] and has continue to facilitate a decrease since then [54]. 

Scientific research is essential to understanding hybridization and the interplay between research and management offers an interesting opportunity to examine this process over the long-term. Initial scientific inquiries after the PHVA were to establish studies to measure, monitor, and manage hybridization in the Recovery Area. In doing so, a complete reconstruction of a red wolf pedigree has been established and this most likely represents the most complete database for any wild population. It's now understood that hybridization between red wolves and coyotes is not directional in terms of the wolves’ sex and hybrids backcross with both species [53,55,56]. Furthermore, current research has identified young, inexperienced red wolves with coyote ancestry to be more likely to breed with coyotes [56]. Despite these successes in measuring and monitoring hybridization, ecological explanations for hybridization have been lacking. In other words, little quantitative information exists on mate selection and possible reproductive barriers between red wolves and coyotes [56,57], and future research efforts should focus on discovering possible reproductive isolating mechanisms that exist between red wolves and coyotes. 

Hybridization between red wolves and coyotes implies the obvious break down of reproductive barriers and the two species consort and breed with one another when situations favor opportunities to mate with congenerics. Currently, no extrinsic reproductive barriers (*i.e.*, geographic barriers) exist between red wolves and coyotes because coyotes are ubiquitous throughout the red wolf's historic range. Hybridization occurs between the two species when a red wolf and a coyote form a breeding pair that will defend a territory together until the death or displacement of a mate. Consequently, the red wolf-coyote pair will produce hybrid offspring and maintain pack dynamics similar to gray wolves [36,58,59], red wolves [9,60,61], and coyotes [62,63,64]. This should be expected because monogamous breeding appears to be a phylogenetic component that operates at the family level and group living is common within *Canis* [65,66]. Therefore, if an isolating mechanism exists, it's most likely to be an intrinsic isolating factor (*i.e.*, behavior) that would prevent pair formation between red wolves and coyotes. Understanding the ecology of red wolf-coyote interactions is crucial to define species traits that serve as isolating mechanisms, describe how these traits prevent hybridization, and identify what selection forces in nature favor the maintenance of red wolves and coyotes as separate species.

If intrinsic isolating factors do exist between red wolves and coyotes, then behaviors that promote sexual isolation of individual red wolves and coyotes should be associated with phenotypes that promote divergence in behavior and genetic discontinuity between the two species. Red wolves and coyotes share the same body plan but do not overlap in body size in which red wolves are the larger species [67]. As a result, body size is the primary trait that distinguishes red wolves from coyotes and it most likely facilitates differential use of resources between the two species. It is well established that body size has a major effect on inter- and intraspecific interactions of mammalian carnivore species in which competitive interactions are strongly asymmetrical with larger species displacing smaller competitors [68,69,70,71]. Furthermore, body size is a key predictor of life history traits, population growth rates, density, space use, and predator-prey dynamics [70,72,73,74,75,76]. It is logical that red wolves and coyotes are not exempt from the broad influences that body-size allometries have at individual-, population-, and community-level processes. Understanding how body size differences lead to differences in red wolf and coyote resource demands, demographics, diet, and space use will lead to more comprehensive understanding of red wolf-coyote interactions and identify what behaviors facilitate genetic discontinuity between the two species.

Recent research has allowed the Recovery Program to measure, monitor, and manage hybridization in NENC. However, preventing hybridization using reproductive sterilization techniques is heavy handed and a short-term strategy to jump start red wolf colonization. There are other important biological considerations to be addressed and research objectives regarding hybridization should shift in the direction of studying the relationship between phenotypic traits and hybridization. For instance, when choosing a mate, do red wolves and coyotes use a criterion of mate quality as a predictor of benefits that potential mates offer and, if so, how does choosing for mate complementarity effect partner fidelity and breeding pair stability? These types of research objectives could associate specific traits with hybridization and breeding success and, eventually, allow biologists to detect selection processes within the red wolf and eastern coyote populations. Reproductive barriers are maintained through ecological, demographic, and developmental conditions [77] and understanding how sexual isolation operates is crucial to the restoration of red wolves.

### 3.2. Inbreeding Effects

Inbreeding can increase the risk of extinction for small populations by decreasing reproductive rates and increasing susceptibility to environmental change and disease [78,79,80,81]. A primary goal of many conservation programs is to minimize inbreeding depression, the deleterious effects of inbreeding, because of the link between increased inbreeding and loss of population viability [82,83,84]. As a small population pushed to the brink of extinction, the red wolf suffered considerable loss of genetic diversity and obviating the potential effects of inbreeding depression and further loss of genetic diversity on red wolf fitness is a conservation goal [7]. Given inbreeding depression may occur when red wolves mate with closely related kin, and as a population founded by few individuals, managing the overall relatedness of captive and wild populations poses challenges for restoration efforts.

Captive breeding of red wolves began three decades ago to preserve the species and provide demographic security. Preservation of genetic diversity in captivity requires using a red wolf Population Analysis and Breeding and Transfer Plan to select sires and dams for artificial breeding [85]. The long-term goal is to preserve 80–90% of the genetic diversity for 150 years [7] and, currently, the captive red wolf population has retained 89.5% of the genetic diversity that existed in the 14 founders [18,85]. Although heritable defects, such as progressive retinal atrophy, malocclusion, and undescended testicles, were observed in a small number of captive red wolves, early studies that examined juvenile survival and litter size reported no observable inbreeding depression in the red wolf captive program [18,86,87]. Subsequent studies found increased levels of inbreeding in the captive population were correlated with decreased litter size, but overall, inbreeding depression was minimal [19]. Rabon and Waddell [19] concluded that improvements in husbandry, veterinary care, and nutrition positively contribute to pup survival and offset the negative effects of inbreeding in the captive population. However, these services are not extended to red wolves in the wild and understanding the effects of inbreeding in the wild population requires further study.

Red wolves are social carnivores in which intraspecific aggression and delayed dispersal play an important role in pack dynamics. The small size of the wild population and the high level of relatedness among individuals increase the risk for incestuous mating to occur. The influence of mate choice and inbreeding avoidance behavior on population dynamics remains poorly understood. It’s been shown that dispersal is an important inbreeding avoidance behavior in other canid species that results in few inbred matings [88,89,90]. Similarly, Sparkman *et al.* [91] found few instances of breeding between 1st degree relatives in wild red wolves and concluded that dispersal behaviors reduced the risk of inbreeding. Red wolf behaviors associated with inbreeding avoidance suggest that inbreeding has a negative effect on fitness and may influence population dynamics.

Inbreeding levels of wild red wolf populations may be high and the effect of inbreeding avoidance on hybridization with coyotes remains unknown. Inbreeding avoidance may cause red wolves to outbreed with a closely related species, such as coyotes, when inbreeding leads to severe fitness consequences. During the mid-2000s, Recovery Program biologists observed dispersing red wolves passing through territories of potentially available red wolf mates and pair-bonding with coyotes. They speculated inbreeding avoidance may influence red wolf mate choice and facilitate hybridization. The premise behind this observation is a hypothesis that assumes when red wolves cannot locate red wolf mates unrelated to them they will opt to breed with unrelated coyotes to avoid incest [92]. Therefore, understanding how inbreeding depression influences hybridization between red wolves and coyotes has become a concern for managing the wild red wolf population.

Research on the effects of inbreeding should involve both ecological and genetic analyses to investigate red wolf and coyote pair formation and how inbreeding avoidance influences hybridization and red wolf fitness. One particular area of promise is sequencing major histocompatibility complex (MHC) genes to examine red wolf kin recognition and mate choice. MHC genes were originally identified in inbred mice during skin graft experiments in which MHC molecules of the host recognized graft tissue as foreign antigens and attacked them [93]. Since then, MHC genes have been discovered to play a critical role in cellular immune response and correlations between MHC alleles, haplotypes, or heterozygosity and pathogen resistance have been shown for a number of species [94,95]. Given that MHC variation affects disease resistance, there may be an advantage to avoid kin and other mates with similar MHC alleles or haplotypes [96,97]. Studies have found MHC-dependent mate choice in both captive and wild species where individuals preferred MHC dissimilar mates [98]. Cooperative group living is a primary adaptive characteristic of red wolves and individuals are likely to recognize kin. How MHC variation influences kin recognition and, subsequently, inbreeding avoidance and hybridization in red wolves is unknown. Red wolves are known to have fewer MHC alleles than other wild canid populations [44] and future research should evaluate how MHC variation may influence mate selection, and hybridization.

### 3.3. Red Wolf Demographics

It's well established that variation in survival and reproduction are responsible for the dynamics of populations [99,100,101,102,103]. Accurate estimates of survival and reproductive rates are essential for conservation programs to minimize extinction risks and promote conditions enhancing the persistence of small, vulnerable populations [104,105]. Population viability analysis (PVA) has traditionally been used to project population trajectories into the future based on ecological and demographic parameters [106,107]. The red wolf population currently exists as a small, vulnerable population with a high risk of extinction to demographic and environmental stochasticity. Understanding how the red wolf population is expected to change in response to environmental conditions is dependent on accurate estimates of vital rates and realistic population estimates from quantitative models.

A primary goal of red wolf recovery is to establish and maintain a red wolf population of 220 individuals in three disjunct populations within the species’ historical range [7]. To evaluate the red wolf population and its viability in the presence of a ubiquitous coyote population, a PVA model was developed at the PHVA to predict population trends and the effect of hybridization on red wolf persistence [16]. The 1999 PVA predicted that red wolves would increase 20% each year for about 10 years before reaching a carrying capacity limit of 140 individuals. Low mortality for wild wolves was assumed to drive the rate of population growth and, despite not reaching 220 individuals, no immediate risk of extinction was suspected given this scenario. When hybridization was incorporated into the 1999 PVA, increased loss of female red wolf breeders to coyote encroachment was predicted to suppress reproductive rates of red wolves to a level too low to offset natural and human-mediated mortality. Therefore, increasing levels of hybridization increases the risk of extinction not only through red wolf assimilation into the coyote population but, also through an inability to replace red wolves lost to mortality.

The 2007 five-year status review (hereafter 2007 Review) of red wolves indicated the NENC population had fluctuated between 80-130 individuals per year since 1999 [18]. With an estimated carrying capacity (*K*) of 140 individuals that was reached in 2001, it was assumed that the red wolf population would continue to expand in subsequent years because red wolves occupied approximately 60% or less of the Albermarle Peninsula land area [18]. However, the red wolf population did not expand but, rather, gradually declined to approximately 100 individuals since peaking in 2001 (see Red Wolf Recovery Program Quarterly Reports). Preliminary analysis of red wolf demographics from 1999 until 2007 indicated overall annual red wolf survival rate was 78.2% and anthropogenic sources of mortality (e.g., gunshots, trapping, and vehicle strikes) accounted for 58% of red wolf deaths [18]. The 2007 Review reported the high proportion of red wolf deaths by anthropogenic factors was additive to other mortality sources and that red wolf fatalities resulting from gunshots remains the most problematic to red wolf persistence.

Red wolves were seven times more likely to be killed during the North Carolina white-tailed deer (*Odocoileus virginianus*) hunting season (October 15–December 15) than during the non-hunting season [18,57]. Illegal take of red wolves is believed to hamper red wolf population growth because it disrupts natural behavioral dynamics that effect demographic processes [108,109]. Furthermore, reduction of red wolves increases coyote presence in the Recovery Area by breaking up packs and destabilizing social dynamics, which reduces the red wolf's ability to hold and defend territories against coyotes. The breeding season for red wolves occurs during white-tailed deer and American black bear (*Ursus americanus*) hunting seasons and increased mortality rates during this time forces red wolf breeders to quickly replace lost mates. When red wolves lost mates to gunshots during the hunting seasons they were more likely to pair with coyotes or fail to replace their mates than to pair with red wolves [18]. Evaluating the breeding records and individual histories of red wolves involved in hybridization events, Bohling [56] found most hybridization events occurred after red wolves lost mates to gunshots and suggested that social structure and stability play a critical role in preventing hybridization. Similarly, Rutledge *et al.* [109] found intense harvest of eastern wolves around Algonquin Provincial Park (APP) during the 1960s to have exacerbated hybridization with coyotes. Therefore, it is prudent for red wolf conservation that managers better understand how high mortality caused by illegal killing of red wolves during the hunting season may disrupt social structures, influence population dynamics, and promote hybridization with coyotes.

The PHVA warned that human-caused mortality that is additive would facilitate hybridization and increase the risk of extinction for red wolves. Therefore, research on red wolf demographics should focus on elucidating mechanisms that influence persistence of wolves on the landscape. This requires use of the Recovery Program's long-term monitoring data of the NENC red wolf population. Long-term monitoring involves annual trapping of red wolves during the fall to radio-tag juvenile and adult red wolves and inspection of dens during the spring to count and transponder pups [51]. These efforts allow the Recovery Program to identify individual red wolves at birth and monitor them until death to collect baseline data on survival and reproduction. Demographic parameters such as survival and population size can be estimated from capture-recapture data [110,111,112] and research efforts should incorporate red wolf monitoring data to develop accurate parameters. Research objectives should estimate annual rates of population change and age-specific survival and reproductive rates of the red wolf population. Additionally, effects of natural and anthropogenic sources of mortality on red wolf persistence should be examined to understand how environmental conditions affect population dynamics over the short- and long-term. This type of research would provide accurate estimates of population parameters for PVAs and assist in developing a valuable framework to evaluate important ecological questions related to red wolf population dynamics. 

## 4. Conclusions

Created in the wake of new societal values, the Red Wolf Recovery Program was tasked by the USFWS with the responsibility of restoring red wolves within their historic range. Along the road to saving the red wolf from extinction, the Recovery Program extirpated the species from the wild to prevent its genetic assimilation into the expanding coyote population. The Recovery Program established a captive-breeding program, and despite starting with 14 founders, grew a captive population of red wolves used for future reintroductions into the wild. During fall of 1987, the red wolf became the first carnivore completely extirpated from the wild to be successfully reintroduced back into its historic range. Today, the Recovery Program manages the only wild population of red wolves on the Albemarle Peninsula of North Carolina. However one views the merits of this effort to restore red wolves, it is a story with challenges and one worth contemplation.

Disagreements about the nature of coyote-like DNA found in red wolves have created controversy in red wolf taxonomy and conservation. The initial discovery of coyote-like haplotypes in red wolves spurred conclusions that the species originated through modern hybridization between gray wolves and coyotes [22,38]. As a result, academic debates during the 1990s focused on the role of modern hybridization in red wolves and its implications for red wolf conservation [15,26,113,114]. However, later research reported the coyote-like DNA found in red wolves indicated a shared ancestry with eastern wolves and coyotes, and concluded that all three species evolved in a New World canid lineage independent of gray wolves [20,23,50]. Predictably, the academic debate has begun shifting towards resolving whether eastern and red wolves are conspecific [20,50,115,116]. Taxonomy is fluid because species evolve and competing concepts over species statuses are not uncommon. In the case of the red wolf, the lack of historic and geographic specimens coupled with modern hybridization between red wolves and coyotes facilitate conflicting conclusions with regards to the species origin. Therefore, it's realistic to expect scientific debate over the taxonomic status of red wolves and, as future studies provide new information, revisions to competing hypotheses regarding species origin should be expected.

Any discussion of red wolf recovery must occur against the backdrop of current ecological and anthropogenic challenges. Although hybridization, inbreeding, and demographics were discussed separately earlier, these three issues are intrinsically related because they are influenced by the presence and management of coyotes. Therefore, these issues are complex and controversial causing management plans to promote recovery efforts to be more difficult than planned. Prior to the mid-1990s, coyotes were rare but increasing in NENC and Recovery Program biologists anticipated eventual colonization of the Peninsula by coyotes. The use of sterilized coyotes as space holders allowed the Recovery Program to saturate the Recovery Area with territories of red wolf packs and sterile coyote pairs. During the early 2000s, most coyotes captured, sterilized, and released with radio-collars failed to establish territories or pair with a space holder. In other words, the Recovery Program effectively saturated the Peninsula with canid territories and coyotes dispersing into the Recovery Area failed to find available space or mates and eventually left. However, legislation (NCGS § 113 273) passed by the NC General Assembly allowing owners of fox pens to buy live coyotes from licensed trappers and hunt them within their fox pens [117] may negatively affect these efforts by disrupting red wolf packs and sterilized coyote space holders. Fox pens are enclosures averaging 250 ha in which gray foxes (*Urocyon cinereoargenteus*), red foxes (*Vulpes vulpes*), and coyotes are hunted with hound dogs for sport in approximately 20 states [118,119,120]. Fox pen operations create legal and illegal markets for the importation and release of coyotes for hunting opportunities, and those markets may supplement local coyote populations through accidental or intentional releases of coyotes into the wild. The number of coyotes trapped in the Recovery Area increased each year after legal trafficking of live coyotes was permitted in 2003 [54]. Although the number of red wolves captured and hunted in fox pens is unknown, disappearance and illegal take of red wolves has increased since the passage of the law. Increased efforts by trappers to capture coyotes and increased vigilance of deer hunters to shoot coyotes have stagnated red wolf population growth by breaking up red wolf packs and removing sterilized coyote space holders from the landscape through accidental and purposeful killing of red wolves and sterilized coyotes [54].

Evident by the widespread persistence and range expansion of coyotes, current policies to control their populations are ineffective and have failed to significantly reduce coyote populations. Laws promoting trafficking and nighttime hunting of coyotes increase the chances that red wolves will be accidentally or purposefully killed by hunters, and attempts to remove these hunting laws are constrained by organized hunting and trapping groups. Increased killing of red wolves by predator-control programs during the early 20th century facilitated the decline of red wolves and promoted their hybridization with expanding coyotes. Recent research showed intense harvest of eastern wolves also facilitated hybridization with coyotes by disrupting the population's social structure [109]. Today, increased killing of red wolves by humans appears to be disrupting red wolf packs and facilitating hybridization with coyotes [56]. Increased relatedness of red wolves through discriminant killing opportunities will eventually lead to inbreeding depression in the wild population. Use of MHC genes to evaluate red wolf mate choice could lead to insights of how red wolves respond reproductively to anthropogenic changes and how MHC variation affects resistance to potential diseases that can be introduced through trafficking coyotes. Therefore, controlling hybridization and inbreeding requires understanding how anthropogenic sources of mortality facilitate conditions favorable to hybridization.

Increased mortality rates of red wolves and coyotes promote high turn-over rates of territories and erode the effectiveness of sterilization methods. Despite this, continued use of sterilization and efforts to increase the number of red wolves on the landscape will likely fail to prevent hybridization if reproductive barriers do not exist in the first place [55]. Key to developing effective management that prevents the hybridization of sympatric red wolf and coyote populations is to identify unique traits of both species that promote sexual isolation. Within the Recovery Area, some individual red wolves and sterile coyotes appear to always prefer conspecifics as mates while others show random preferences, and assortative mating within both populations may indicate an intrinsic reproductive barrier. In the hybridization section of this paper, we discussed evaluating the effects of body size on mate complementarity between red wolves and coyotes because body size is the most characteristically distinct trait between the two species. Life history traits (*i.e.*, rates of individuals growth, reproduction, and mortality), population-level processes (*i.e.*, abundance and space use), and community-level interactions (*i.e.*, predator-prey dynamics and interspecific interactions) are known to correlate with body-size allometries regardless of taxonomic status [75,121,122,123]. Therefore, there are compelling reasons to study how phenotypes facilitate the outcomes of red wolf-coyote interactions because phenotypes are the direct interface between the two species. Selection acts directly on phenotypes with genetic change occurring as an indirect consequence and phenotypes have ecological effects on population dynamics and community structure [124,125,126,127,128,129]. If certain phenotypes serve as reproductive barriers between red wolves and coyotes, management can manipulate selection to achieve desired demographic effects and reduce hybridization.

A major impediment to red wolf restoration is the limited knowledge about traits that facilitate behavioral and ecological differences between red wolves and coyotes. This is critical to red wolf restoration because expanding our knowledge about mechanisms that facilitate stable and reproductively isolated red wolf populations will allow us to recognize responses of red wolves to changing environments. This knowledge guides research to make accurate inferences and predictions about the future and promotes implementation of appropriate management. The reality of incomplete reproductive isolation may present challenges to red wolf restoration but evolution is ongoing and management efforts should promote conditions that allow for the gradual evolution of reproductive barriers. Although much work remains to be done, information and experiences gained from more than 25 years of restoration efforts have made crucial contributions to the future of the red wolf. They also allow us to formulate areas of investigation that are of direct relevance to the restoration of red wolves.
